# Acetyl‐11‐keto‐β‐boswellic acid ameliorates renal interstitial fibrosis via Klotho/TGF‐β/Smad signalling pathway

**DOI:** 10.1111/jcmm.13766

**Published:** 2018-07-28

**Authors:** Minna Liu, Tianlong Liu, Peijin Shang, Yikai Zhang, Limin Liu, Ting Liu, Shiren Sun

**Affiliations:** ^1^ Department of Nephrology Xijing Hospital Fourth Military Medical University Xi'an China; ^2^ State Key Laboratory of Cancer Biology Fourth Military Medical University Xi'an China; ^3^ Department of Pharmacy Xijing Hospital Fourth Military Medical University Xi'an China; ^4^ Medical Equipment Quality Supervision and Inspection Institute Shaanxi Food and Drug Administration Xianyang China; ^5^ Department of Nephrology the Fourth Hospital of Xi'an Xi'an China

**Keywords:** Acetyl‐11‐keto‐β‐boswellic acid, HK‐2 cells, Klotho/TGF‐β/Smad signalling pathway, renal interstitial fibrosis, unilateral ureteral obstruction

## Abstract

Acetyl‐11‐keto‐β‐boswellic acid (AKBA), an active triterpenoid compound from the extract of Boswellia serrate, has been reported previously in our group to alleviate fibrosis in vascular remodelling. This study aimed to elucidate the in vivo and in vitro efficacy and mechanism of AKBA in renal interstitial fibrosis. The experimental renal fibrosis was produced in C57BL/6 mice via unilateral ureteral obstruction (UUO). Hypoxia‐induced HK‐2 cells were used to imitate the pathological process of renal fibrosis in vitro. Results showed that the treatment of AKBA significantly alleviated UUO‐induced impairment of renal function and improved the renal fibrosis by decreasing the expression of TGF‐β1, α‐SMA, collagen I and collagen IV in UUO kidneys. In hypoxia‐induced HK‐2 cells, AKBA displayed remarkable cell protective effects and anti‐fibrotic properties by increasing the cell viability, decreasing the lactate dehydrogenase (LDH) release and inhibiting fibrotic factor expression. Moreover, in obstructed kidneys and HK‐2 cells, AKBA markedly down‐regulated the expression of TGFβ‐RI, TGFβ‐RII, phosphorylated‐Smad2/3 (p‐Smad2/3) and Smad4 in a dose‐dependent fashion while up‐regulated the expression of Klotho and Smad7 in the same manner. In addition, the effects of AKBA on the Klotho/TGF‐β/Smad signalling were reversed by transfecting with siRNA‐Klotho in HK‐2 cells. In conclusion, our findings provide evidence that AKBA can effectively protect kidney against interstitial fibrosis, and this renoprotective effect involves the Klotho/TGF‐β/Smad signalling pathway. Therefore, AKBA could be considered as a promising candidate drug for renal interstitial fibrosis.

## 1 INTRODUCTION

Renal interstitial fibrosis, characterized by excessive accumulation of extracellular matrix (ECM), is the final common pathway of chronic kidney disease (CKD) progress to end‐stage renal disease (ESRD), irrespective of the initiating pathology.^1^ Inhibiting the progression of interstitial fibrosis is considered as a potential strategy to protect the kidney from the deleterious effects of CKD. Numerous studies have revealed that renal interstitial fibrosis is mediated by multiple mechanisms, and the central cellular event is the accumulation of myofibroblasts, which are primarily responsible for the deposition of ECM at pathologic conditions.^2^ Persistent production of ECM decreases the glomerular infiltration rate and renal function, eventually resulting in the acceleration of renal injury.^3^

Klotho is a membrane‐bound protein abundantly expressed in the kidney, and which was recognized as a promising protein uniquely poised to provide the basis for anti‐fibrotic treatment strategies.^4^ Numerous studies have indicated that Klotho deficiency induces and promotes tubulointerstitial fibrosis progression, and overexpression of Klotho protects against renal fibrosis.^5^, ^6^ In addition, Klotho was reported to inhibit transforming growth factor‐β1 (TGF‐β1) signalling by directly binding to TGFβ‐RII, this is a vital mechanism for Klotho‐mediated prevention of renal fibrosis.^7^ TGF‐β1 has been identified as the key profibrogenic cytokine via activating downstream mediators, especially Smad proteins, and finally regulating ECM production.^8^ Thus, blocking the Klotho/TGF‐β/Smad signalling pathway may be promising for preventing renal fibrosis and preserving renal function.

**Figure 1:**
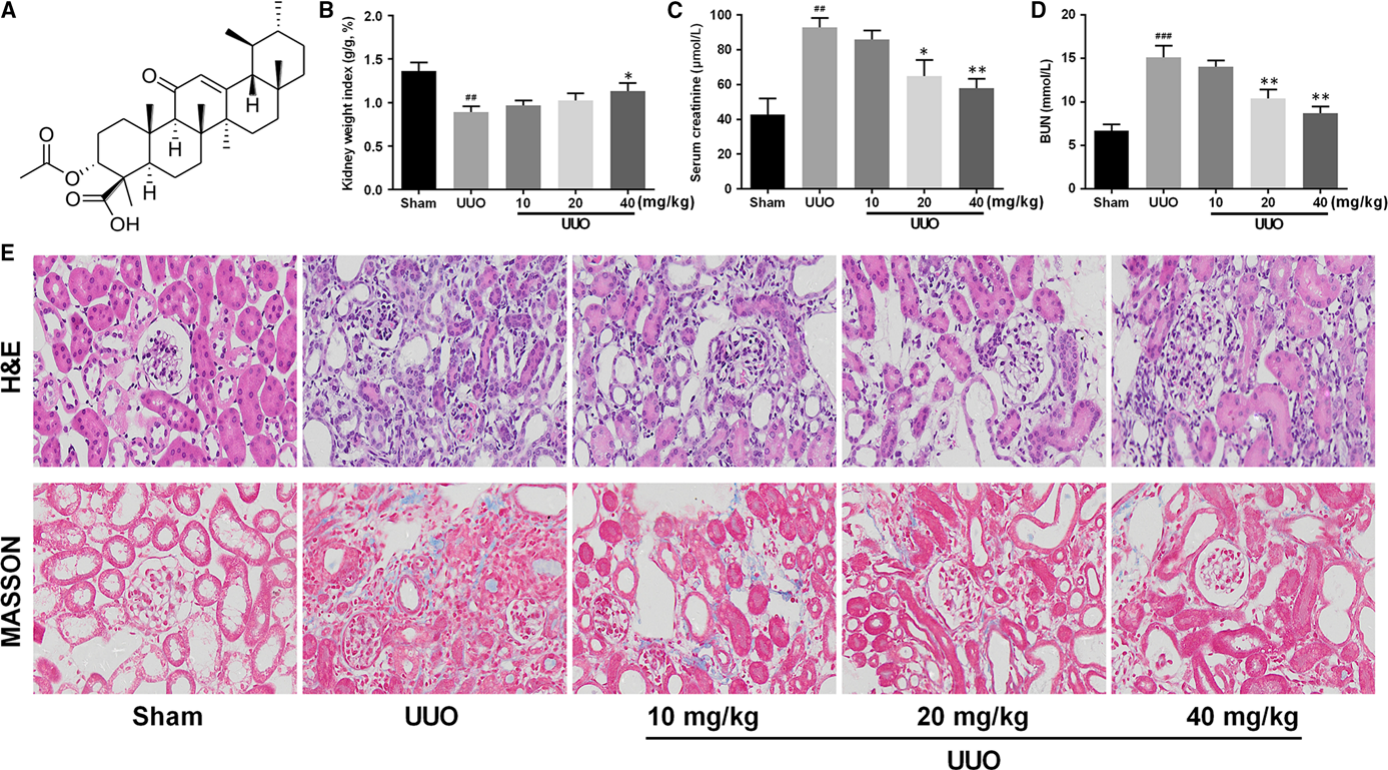
Effect of AKBA on renal injury induced by unilateral ureteral obstruction (UUO). A, Chemical structure of AKBA. B, Kidney weight index was evaluated by the percentage of obstructed kidney/body weight in each mouse. C, The Scr level of mice 14 d after UUO (n = 8). D, The BUN level of mice 14 d after UUO. E. H&E and MASSON staining of kidney tissue. Data were presented as the mean ± SD (n = 8). ^##^ *P* < .01, ^###^ *P* < .001 vs sham group; *P < .05, **P < .01 vs UUO group

Acetyl 11‐keto‐β‐boswellic acid (AKBA), a pentacyclic triterpenoid compound (Figure 1A), is one of the most potent active principles within the multi‐component mixture of Boswellia serrata resin. Boswellia serrata resin extracts (Boswellic acids) has been reported to have significant anti‐fibrosis activity in many conditions, including hepatic fibrosis,^9^ colonic fibrosis^10^ and pulmonary fibrosis.^11^ The anti‐renal fibrosis property of pentacyclic triterpenoid has attracted increasing attention recently. For example, betulinic acid ameliorates experimental diabetic renal fibrosis by inhibiting the activation of NF‐kB signalling pathway.^12^ Oleanolic acid, the similar pentacyclic triterpene compound, exerts its therapeutic effect on tubulointerstitial fibrosis in chronic cyclosporine nephropathy.^13^ Another study revealed that Asiatic acid attenuates renal fibrosis by rebalancing TGF‐β/Smad signalling pathway.^14^ More encouragingly, previous studies of our group have found that AKBA is beneficial for vascular remodelling and fibrosis by blocking TGF‐β/Smad pathway. This prompted us to address in greater detail the role and mechanism of AKBA in renal fibrosis.

In this study, we applied in vivo and in vitro renal fibrosis paradigms to analyse the protective properties of AKBA. We hypothesized that AKBA would provide reno‐protection against fibrosis in UUO‐induced mice and in hypoxia‐induced HK‐2 cells, and that the protective effects were exerted through inhibiting the Klotho/TGF‐β/Smad pathway.

## 2 MATERIALS AND METHODS

### 2.1 Animals

Male C57BL/6 mice (6‐8 weeks, 18‐20 g) were supplied by Medical Laboratory Animal Center, Fourth Military Medical University (Xi'an, China). All experiments were conducted in adherence with the National Institutes of Health Guidelines for the Use of Laboratory Animals and have been approved by the Fourth Military Medical University Committee on Animal Care. The mice were kept in plastic cages at 22 ± 2°C under a 12 hours light/dark cycle, with free access to pellet food and water. An acute oral toxicity study of AKBA on normal rats has been conducted in our previous research, and the results demonstrated that AKBA did not show any sign of toxicity or abnormal symptom in the rats of dosing up to 1600 mg/kg. Therefore, the dosage of AKBA in this study is considered to be safe.

### 2.2 Experimental protocols

After 7 days of acclimatization, the animals were randomly divided into 5 groups (n = 8 in each group): sham‐operated group (Sham), UUO‐operated group (UUO), low AKBA dosage group (10 mg/kg/d), middle AKBA dosage group (20 mg/kg/d) and high AKBA dosage group (40 mg/kg/d). The UUO operation was produced as described previously.^15^ Briefly, the mice were anaesthetized with 1.5% sodium pentobarbital (50 mg/kg), and then the left ureter was separated and ligated with 4‐0 silk sutures. The remnant ureter between the ligatures was cut‐off. Sham group underwent the same procedure and exposured the left ureter without ligation. After completion of modelling, the mice were administered with different dosage of AKBA or equal volumes of sterile saline by gavage. At 14 days after treatment, the mice were weighed and killed. Blood was collected for biochemical examination, and the obstructed kidneys were harvested and weighed for further researches.

### 2.3 Renal function test

The blood samples were centrifuged to obtain serum for evaluation of serum creatinine (Scr) and blood urea nitrogen (BUN). A Creatinine Assay Kit and Urea Assay Kit (Jiancheng Biotechnology, Nanjing, China) were used for Scr and BUN test according to the manufacturer's instructions.

### 2.4 Histology and immunohistochemistry

Kidneys were fixed with 4% paraformaldehyde for at least 24 hours, and then they were dehydrated, embedded and cut into 5‐μm sections. The sections were stained with haematoxylin & eosin (H&E) and Masson's trichrome for general morphological assay.

Immunohistochemical detection was conducted with general approaches. Anti‐α‐SMA (1:500, CST, Boston, MA, USA, #56856), anti‐TGF‐β1 (1:200, Abcam, Cambridge, UK, #92486) and anti‐Smad2/3 (1:200, Santa Cruz, Santa Cruz, CA, USA, #398844) were used as the primary antibodies. Image Pro Plus 6.0 (Media Cybernetics, Inc., Rockville, MD, USA) image analysis software was used to calculate the mean optical density value. At least 5 non‐overlapping cortical fields per section were analysed for each animal in a blinded manner.

### 2.5 Cell culture and treatment

Human proximal tubular epithelial cell line (HK‐2) was obtained from Type Culture Collection of the Chinese Academy of Sciences (Shanghai, China). HK‐2 cells were cultured in DMEM/F12 (Gibco, Grand Island, NY, USA) medium containing 10% foetal bovine serum (FBS) under normoxic conditions (21% O_2_, 5% CO_2_, 37°C). Cells were seeded in 6‐well plates at 1 × 10^6^ cells/mL and divided into 5 groups: control group (normoxic‐treated), hypoxia group (1% O_2_, 5% CO_2_, 37°C), hypoxia + 10 μmol/L AKBA, hypoxia + 20 μmol/L AKBA and hypoxia + 40 μmol/L AKBA. AKBA treatment was conducted 30 minutes prior to hypoxia stimulation and the protein expression was measured 48 hours after the stimulation.

### 2.6 siRNA transfection

To knock down the expression of Klotho, 1 × 10^6^ HK‐2 cells were transfected with siRNA‐Klotho or siRNA‐Negative control (siRNA‐NC) from GeneChem Co., Ltd. (Shanghai, China). The sequence of siRNA‐Klotho is GCAGAATTACATAAACGAA. Transient siRNA transfection was performed with Lipofectamine 3000 transfection reagent according to the manufacturer's instructions. After 24 hours of transfection, the cells were treated with hypoxic stimulation with or without 40 μmol/L AKBA for another 48 hours.

### 2.7 Cell viability

Cell viability was determined at 48 hours post‐hypoxia by 2 widely accepted assays, MTT assay and LDH release assay. The experiments were conducted according to the manufacturer's protocol of a cell cytotoxicity assay kit and a LDH assay kit (Jiancheng Biotechnology).

### 2.8 Western blotting analysis

Total proteins were extracted from the kidneys or HK‐2 cells and the concentration was measured by BCA protein assay kit (Beyotime, Shanghai, China). Western blot assay was performed with the standard method. Briefly, proteins were separated via sodium dodecyl sulfate polyacrylamide gel electrophoresis (SDS‐PAGE). PVDF membrane (Millipore, Bedford, MA, USA) was used to electro‐transfer. After blocking with 5% skim milk, the membrane was incubated overnight at 4°C with primary antibodies: anti‐Klotho (1:500, Abcam, #203576), TGF‐β1 (1:1000, Abcam, #92486), TGFβ‐RI (1:800, Abcam, #31013), TGFβ‐RII (1:1000, Abcam #186838), α‐SMA (1:1000, CST, #56856), Collagen I (1:2000, Abcam, #34710), Collagen IV (1:1500, Abcam, #6586), Smad 2/3 (1:1000, Santa Cruz, #398844), p‐Smad 2/3 (1:800, Abcam, #63399), Smad 4 (1:1000, CST, #38454) and Smad 7 (1:1000, Santa Cruz, #365846), followed by incubation with secondary antibody at room temperature for 1 hour. Glyceraldehyde 3‐phosphate dehydrogenase (GAPDH) was used as the internal control. Protein bands were visualized by enhanced chemiluminescence (ECL) reagent and exposed using BioImaging Systems (UVP, Upland, CA, USA). The relative protein levels were quantified using the Image J software (National Institutes of Health, Montgomery, MD, USA). All the assays were performed in triplicate.

### 2.9 Quantitative RT‐PCR

Total RNA in HK‐2 cells was extracted using Trizol Reagent (Pufei, Shanghai, China) according to the manufacturer's protocol. Quality of RNA was examined and all has reached the request of PCR. About 1 μg of total RNA was processed for cDNA synthesis using a cDNA first‐strand synthesis system (Fermentas International Inc, Burlington, ON, Canada). Amplification and detection were performed with the ABI 7500 Real‐Time PCR system (Applied Biosystems, Foster City, CA, USA). The sequences of the primers used in this study were listed in Table S1. The PCR cycling conditions were as follows: 94°C for 15 minutes to preincubation and for 10 seconds to denaturation, and at 60°C for 30 seconds for 45 cycles to extension. All the assays were performed in triplicate. Transcript levels were calculated using the $2^{-\Delta \Delta C_{t}}$ method and were normalized to GAPDH.

### 2.10 Immunofluorescence staining

HK2 cells were fixed in 4% paraformaldehyde and subsequently permeabilized with 0.1% Triton X‐100. After blocking with 5% BSA at room temperature for 1 hour, cells were incubated with anti‐TGF‐β1 (1:100), anti‐α‐SMA (1:100) and anti‐Collagen IV (1:200) primary antibodies at 4°C overnight. The cells were then incubated with corresponding secondary antibodies and counterstained with DAPI. The stained cells were visualized under a confocal microscope (Nicon C2, Japan). Images were quantified in a blinded fashion.

### 2.11 Statistical analysis

The SPSS (IBM, Armonk, New York, USA) 19.0 was used for statistical analysis. Data were presented as the mean ± standard deviation (SD). Statistical significance was determined using the one‐way analysis of variance (ANOVA) followed by Tukey's multiple‐comparison test. *P* < 0.05 was considered as statistical significance.

## 3 RESULTS

### 3.1 AKBA alleviated renal function and renal interstitial damage in UUO mice

As shown in Figure 1B, the UUO operation significantly reduced the kidney weight index (percentage of obstructed kidney/body weight). After 10 and 20 mg/kg AKBA treatment, the kidney index had no significance, while 40 mg/kg AKBA treatment caused a slight but significant increase of the value. The effect of AKBA on renal function was evaluated by serum creatinine (Scr) and BUN test. Results showed that the levels of Scr and BUN markedly increased after UUO and decreased by 20 and 40 mg/kg AKBA treatment. There's no significance between 10 mg/kg AKBA and UUO group (Figure 1C‐D).

Haematoxylin & eosin staining was used to observe the morphology changes in obstructed kidneys (Figure 1E). Sham‐operated kidney presented normal tubule interstitium, while mice in UUO group showed a significant tubulointerstitial injury, including interstitial expansion, epithelial cells atrophy, inflammatory cell infiltration, glomerular sclerosis and ECM deposition. These injuries were ameliorated by incremental doses of AKBA treatment. Renal interstitial fibrosis level was also evaluated by Masson staining. Increased collagen deposition and area of interstitial fibrosis were observed in the UUO group. However, administration of AKBA markedly decreased the degree of interstitial fibrosis (Figure 1E).

### 3.2 AKBA decreased the expression of fibrotic factors in UUO mice

**Figure 2:**
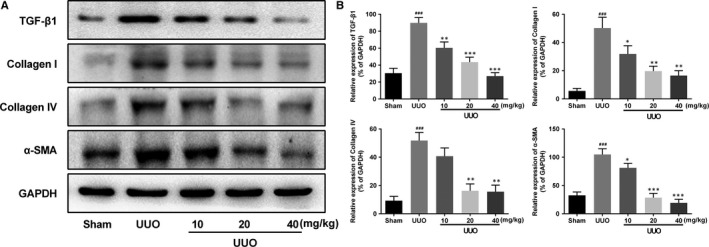
Effect of AKBA on the expression of fibrotic factors in UUO mice. A, Representative photographs showing the expressions of TGF‐β1 collagen I, collagen IV and α‐SMA. B, Quantitative analysis of the optical density in part A. Data were presented as mean ± SD for 3 independent experiments (n = 8). ^###^ *P* < .001 vs sham group; *P < .05, **P < .01, ***P < .001 vs UUO group

Based on the Masson staining above, effect of AKBA on interstitial fibrosis was further valued by detecting the expression of fibrotic factors. TGF‐β1 is considered the vital pro‐fibrotic growth factor in the pathogenesis of interstitial fibrosis. Myofibroblasts play a critical role in fibrosis, thus as a marker of myofibroblast, the expression of α‐SMA was also examined. As shown in Figure 2, compared to the sham group, the expressions of TGF‐β1 and α‐SMA were significantly increased in the UUO kidneys. While after AKBA treatment, the expressions of TGF‐β1 and α‐SMA were effectively reduced in a dose‐dependent manner. Similar tendency was also observed in collagen I and collagen IV expression, as determined by Western blotting. These results indicated that AKBA could attenuate UUO‐induced renal interstitial fibrosis.

### 3.3 AKBA inhibited the Klotho/TGF‐β/Smad signalling pathway in UUO mice

**Figure 3:**
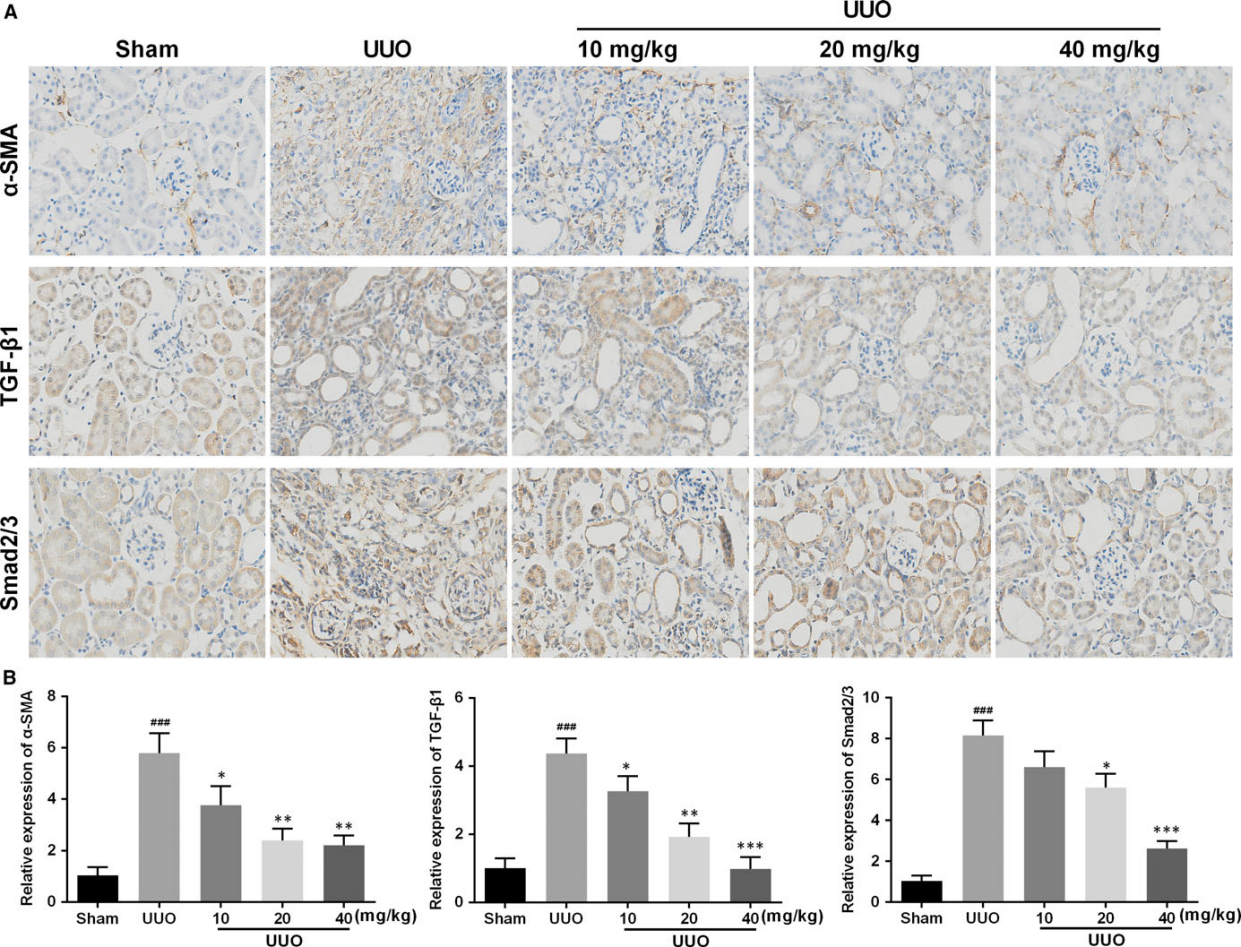
Effect of AKBA on the expressions of α‐SMA, TGF‐β1 and Smad2/3. A, Representative photomicrographs of α‐SMA, TGF‐β1 and Smad2/3 immunohistochemistry on the kidney sections. B, Quantitative analysis of the optical density. Data were presented as mean ± SD (n = 8). ^###^ *P* < .001 vs sham group; *P < .05, **P < .01, ***P < .001 vs UUO group

To investigate the possible mechanisms underlying the effects of AKBA, the Klotho/TGF‐β/Smad signalling pathway was checked in the obstructed kidney. The immunohistochemistry results showed that the overexpression of α‐SMA and TGF‐β1 induced by UUO was significantly restrained by AKBA. Smad2/3, the core protein in Klotho/TGF‐β/Smad pathway, was up‐regulated in obstructed kidneys and decreased after treatment of AKBA (Figure 3).

**Figure 4:**
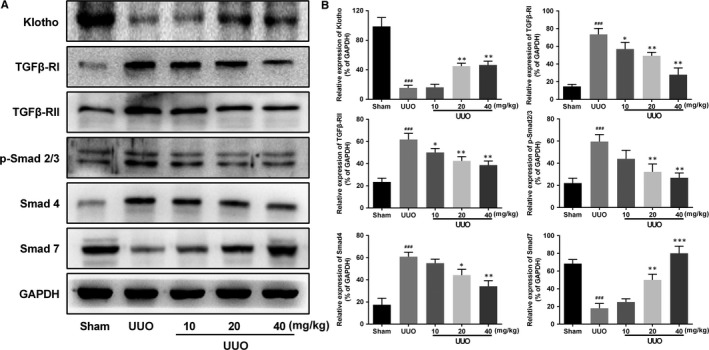
Effect of AKBA on the activation of Klotho/TGF‐β/Smad pathway in UUO mice. A, Representative photographs showing the expressions of TGFβ‐RI, TGFβ‐RII, p‐Smad2/3, Smad4 and Smad7. B, Quantitative analysis of the optical density in part A. Data were presented as mean ± SD for 3 independent experiments (n = 8). ^##^ *P* < .01, ^###^ *P* < .001 vs sham group; *P < .05, **P < .01, ***P < .001 vs UUO group

Next, the exact mechanism was studied by Western blotting. Fourteen days after UUO, the expression of Klotho significantly declined in obstructed kidneys, while the level elevated with AKBA treatment. Besides, the expressions of TGFβ‐RI, TGFβ‐RII, phosphorylated‐Smad2/3 (p‐Smad2/3) and Smad4 were obviously increased compared to the sham group. After treatment of AKBA in low‐to‐high concentration, these elevated protein levels were significantly inhibited. In addition, AKBA dose‐dependently increased the down‐regulation of Smad7 induced by UUO (Figure 4).

### 3.4 AKBA enhanced the viability and decreased the LDH release in hypoxia‐induced HK‐2 cells

**Figure 5:**
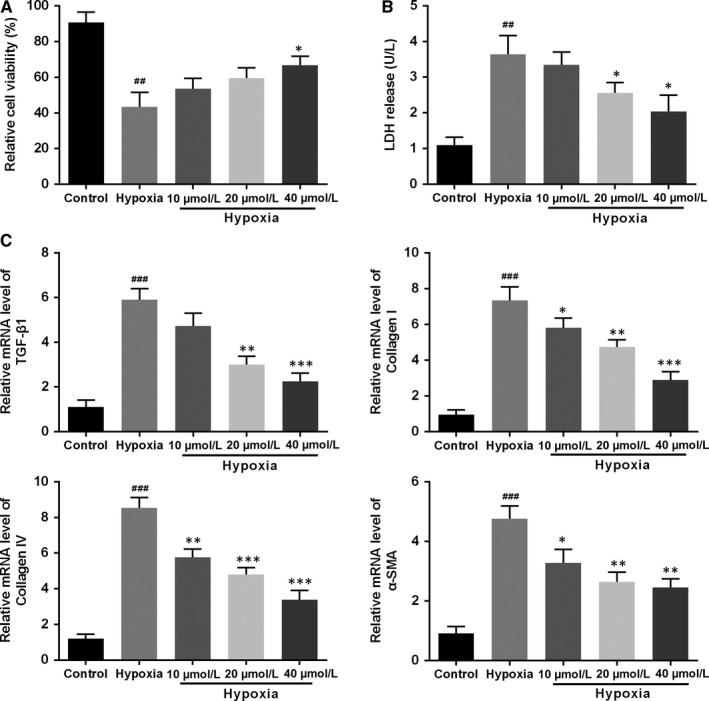
Effect of AKBA on the cell viability and fibrosis level in hypoxia‐induced HK‐2 cells. A, Cell viability of HK‐2 cell 48 h after hypoxia. B, The LDH release of HK‐2 cell 48 h after hypoxia. C, The mRNA levels of TGF‐β1, collagen I, collagen IV and α‐SMA were determined by quantitative RT‐PCR. Data were presented as mean ± SD (n = 8). ^##^ *P* < .01, ^###^ *P* < .001 vs control group; *P < .05, **P < .01, ***P < .001 vs hypoxia group

Emerging evidence has suggested that chronic hypoxia can trigger kidney damage response and cause irreversible pathological change in tubular epithelial cells, including necrosis, apoptosis and phenotype transformation, leading to renal interstitial fibrosis.^16^ Therefore, the cell viability of HK‐2 cells was firstly evaluated at 48 hours after hypoxia exposure in the absence and presence of AKBA treatment. The data revealed that the cell viability was significantly decreased and the LDH release was greatly increased in hypoxia‐induced HK‐2 cells compared with the control group (Figure 5A‐B). When treated with AKBA, the cells MTT value was increased and LDH release was reduced compared with the vehicle treatment under hypoxia condition.

### 3.5 AKBA prevented fibrosis in hypoxia‐induced HK‐2 cells

To further confirm the anti‐fibrosis effect of AKBA on hypoxia‐induced HK‐2 cells, fibrosis‐related factors, including TGF‐β1, collagen I, collagen IV and α‐SMA, were detected by quantitative real‐time RT‐PCR. We found that their levels were routinely increased under hypoxic condition but were obviously reduced after AKBA treatment (Figure 5C).

**Figure 6:**
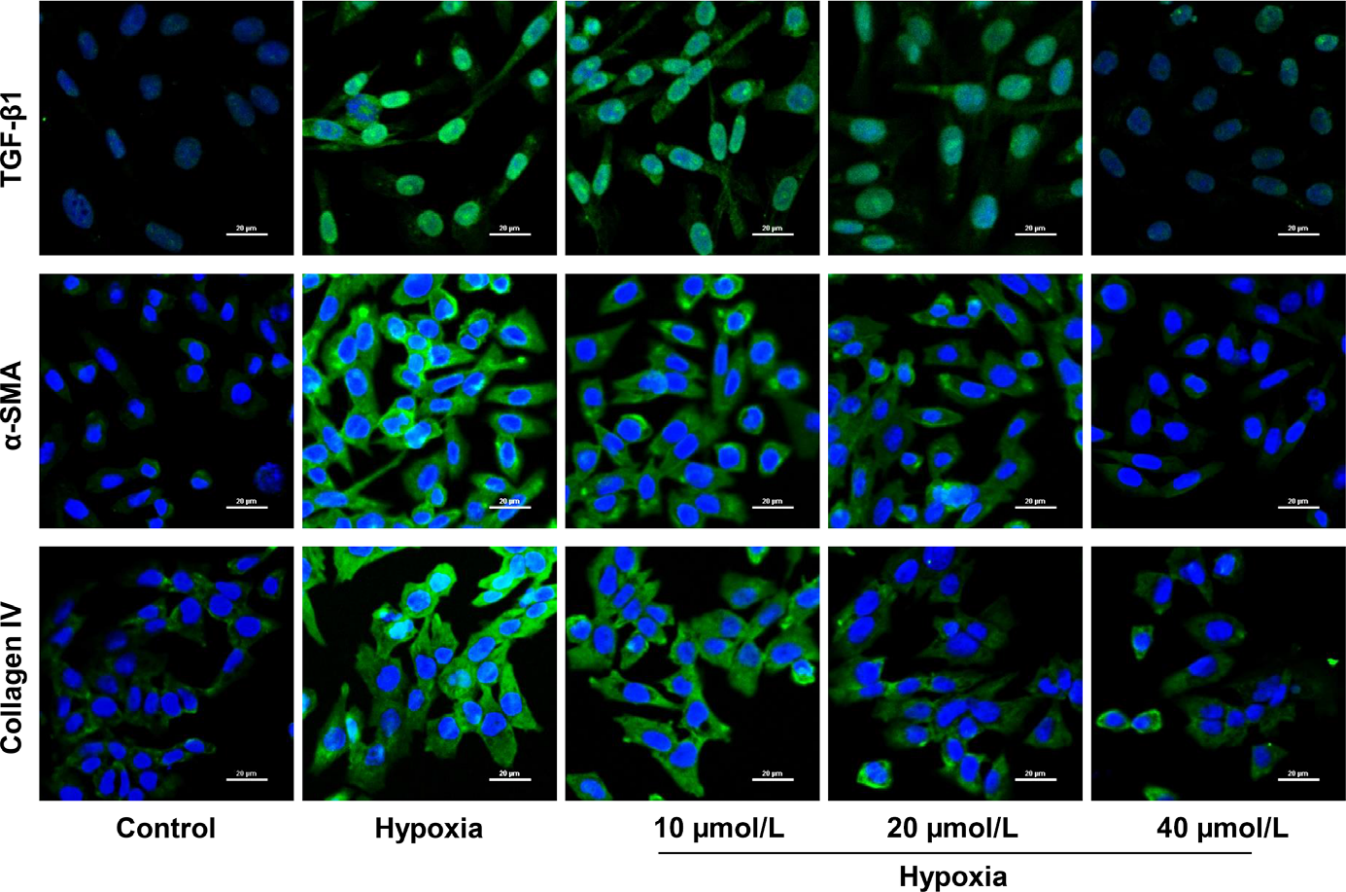
Effect of AKBA on the expressions of TGF‐β1, α‐SMA and collagen IV in hypoxia‐induced HK‐2 cells detected by immunofluorescence. The blue staining refers to the DAPI while the red staining refers to the specific proteins. Scale bar = 20 μm

Likewise, immunofluorescence results manifested that hypoxia could increase the expression of TGF‐β1, α‐SMA and Collagen IV. In comparison with hypoxia group, a noticeable reduction in TGF‐β1, α‐SMA and Collagen IV expression was observed in AKBA treated groups (Figure 6), which suggested that AKBA can effectively relieve fibrosis in hypoxia‐induced HK‐2 cells.

### 3.6 AKBA blocked the Klotho/TGF‐β/Smad signalling pathway in hypoxia‐induced HK‐2 cells

**Figure 7:**
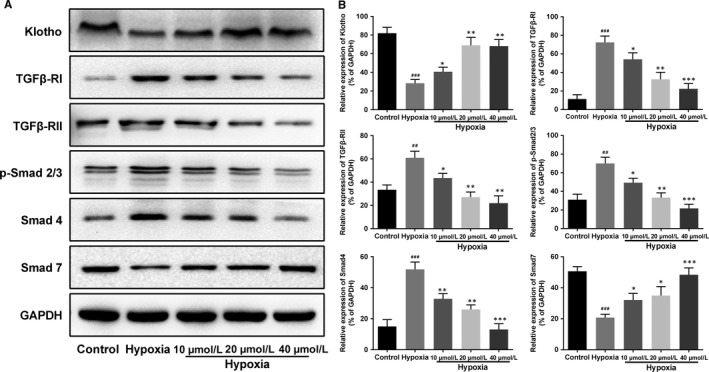
Effect of AKBA on the activation of Klotho/TGF‐β/Smad pathway in hypoxia‐induced HK‐2 cells. The expressions of Klotho, TGFβ‐RI, TGFβ‐RII, p‐Smad2/3, Smad4 and Smad7 were investigated by Western blot. Data were presented as mean ± SD for 3 independent experiments (n = 8). ^##^ *P* < .01, ^###^ *P* < .001 vs control group; *P < .05, **P < .01, ***P < .001 vs hypoxia group

Followed, effect of AKBA on the activation of Klotho/TGF‐β/Smad pathway in vitro was investigated. As expected, expressions of TGFβ‐RI, TGFβ‐RII, p‐Smad2/3 and Smad4 in HK‐2 cells were up‐regulated while the expressions of Klotho and Smad7 were down‐regulated under hypoxia condition. Similar to the study in vivo, AKBA dose‐dependently blocked the activation of Klotho/TGF‐β/Smad signalling pathway in HK‐2 cells (Figure 7).

### 3.7 siRNA‐Klotho reversed the effects of AKBA on the Klotho/TGF‐β/Smad signalling pathway in hypoxia‐induced HK‐2 cells

**Figure 8:**
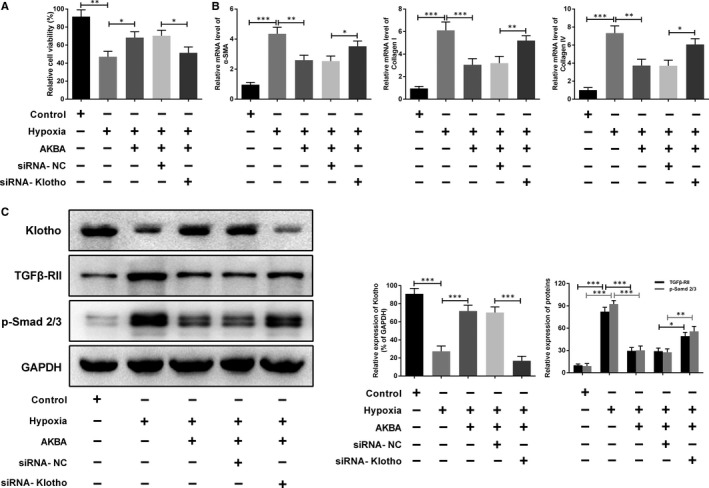
Effect of siRNA‐Klotho on the anti‐fibrosis function and mechanism of AKBA in hypoxia‐induced HK‐2 cells. A, Cell viability of HK‐2 cells. B, The mRNA levels of collagen I, collagen IV and α‐SMA were determined by quantitative RT‐PCR. C, The expressions of Klotho, TGFβ‐RII and p‐Smad2/3 were investigated by Western blot. Data were presented as mean ± SD (n = 8). *P < .05, **P < .01, ***P < .001

To validate the effect of AKBA on the Klotho/TGF‐β/Smad signalling pathway, siRNA was used to knock down the upstream gene Klotho in HK‐2 cells before the hypoxia and AKBA treatment. The results indicated that AKBA markedly increased the cell viability and inhibited the expressions of fibrosis‐related factors. The renal protective effects of AKBA, however, were cut‐off when the cells were transfected with siRNA‐Klotho (Figure 8A,B). Also, the knockdown of Klotho reversed the effect of AKBA on the Klotho/TGF‐β/Smad signalling pathway in hypoxia‐induced HK‐2 cells (Figure 8C). All these results suggested that AKBA ameliorated renal interstitial fibrosis via Klotho/TGF‐β/Smad signalling pathway.

## 4 DISCUSSION

In the present study, we investigated the effect of AKBA on UUO‐induced renal interstitial fibrosis for the first time. The result showed that AKBA plays a markedly protective role against renal injury via anti‐fibrosis effects mediated by the Klotho/TGF‐β/Smad pathway. The renal protective effect and the potential mechanism of AKBA were further confirmed in hypoxia‐induced HK‐2 cells.

Unilateral ureteral obstruction is a classic model for renal interstitial fibrosis research,^17^ renal hemodynamic and metabolic markedly changes after ureteral obstruction, and these sustained changes lead to the transformation of interstitial fibroblasts and excess deposition of EMC. In our results, significant disruption of the renal function indices was observed in UUO kidneys by detecting the kidney/body weight, BUN and Scr values. Meanwhile, renal injury and interstitial fibrosis were found in the histopathological sections after UUO. Whereas AKBA treatment improved the renal function and decreased the pathological injury.

Increasing evidence showed that chronic hypoxia is an important driving factor of renal interstitial fibrosis and leads to end‐stage renal failure.^16^, ^18^, ^19^ Thus hypoxia‐induced HK‐2 cell is often used to imitate the pathological process of renal interstitial fibrosis in vitro.^20^, ^21^ The data revealed that the cell viability was significantly decreased and the LDH release was greatly increased in hypoxia‐induced HK‐2 cells as compared to control group. While the AKBA treatment substantially increased the cell viability and reduced LDH release. These results indicated that AKBA is capable of protecting kidneys from progress to interstitial fibrosis.

Klotho is a multifunctional protein that is well known for its anti‐ageing property. Deficiency of Klotho in mice often leads to short lifespan, cognitive dysfunction, osteoporosis, vascular calcification, etc.^22^ While overexpression of Klotho could extend lifespan^23^ and alleviate renal disease.^24^, ^25^ It was reported that full Klotho knockout mice (Klotho^−/−^) have a more striking phenotype and serious pathological changes than heterozygous Klotho mice (Klotho^+/−^).^4^ Compared to the wild‐type mice, constitutive overexpression of Klotho in mice could lead to less expression of fibrosis‐related proteins, such as α‐SMA and collagen I.^25^ As expected, the results in our study showed that the levels of Klotho were significantly decreased in obstructed kidneys and hypoxia‐induced HK‐2 cells. AKBA administration up‐regulated the expression of Klotho in a dose‐dependent manner. And the renal protective effect of AKBA was reversed with the transfection of siRNA‐Klotho.

As a downstream molecule directedly regulated by Klotho, TGF‐β1 has been extensively recognized as a critical mediator in interstitial fibrosis.^26^ Accumulating evidence has demonstrated that TGF‑β1 is excessively produced by fibroblasts in various kidney diseases with interstitial fibrosis.^27^, ^28^ The activated TGF‐β1 regulates cellular differentiation, proliferation and migration, as well as the protein expression of ECM.^29^ Overexpression of TGF‐β1 could cause renal fibrosis while inhibition of that would improve renal fibrotic lesions.^30^ Our study indicated that the protein expression of TGF‐β1 was increased in UUO kidney, and AKBA could attenuate the elevated TGF‐β1 level in a dose‐dependent manner. In addition, similar effect was observed in HK‐2 cells. As the major components of ECM, α‐SMA, collagen I and collagen IV are tightly bound to renal fibrosis. The synthesis and degradation of these proteins are in a state of dynamic balance under normal physiological condition.^8^ Our results showed that the expression of α‐SMA, collagen I and collagen IV were evidently increased in obstructed kidneys and hypoxia‐induced HK‐2 cells. All the elevated expressions were reversed after AKBA treatment, indicating the anti‐fibrotic role of AKBA.

Among the downstream signalling pathways involving in TGF‐β‐dependent renal interstitial fibrosis, Smads pathway is considered as the most critical one.^31^ TGF‐β1 signal is transduced through its cell membrane receptors I and II (TGFβ‐RI and TGFβ‐RII). Upon TGF‐β stimulating, receptors become activated and trigger the phosphorylation of its downstream signalling molecules: Smad2 and Smad3. Phosphorylated Smad2/3 heteroligomerizes with Smad 4, a common partner Smad, and the complexes translocate into the nucleus to regulate the transcription of target genes.^32^ In a rat UUO model, TGFβ‐RII mRNA significantly increased, and inhibition of TGFβ‐RII ameliorated the progression of renal fibrosis.^33^ Consistent with the reported data, we also found that TGFβ‐RI and RII were highly expressed in the fibrotic kidneys. However, they were markedly inhibited after AKBA treatment. In the context of renal interstitial fibrosis, Smad2/3 was reported to be strongly activated in various CKDs.^34^ In addition, deletion of Smad3 in mice or use of Smad3 inhibitor suppressed the progress of renal fibrosis.^35^, ^36^ As expected, excessive expression of Smad 2/3 was observed both in obstructive kidneys and hypoxia‐insulted HK‐2 cells. After AKBA in low‐to‐high concentrations were given, protein level of Smad2/3 in the treatment group was dose‐dependently decreased. Smad7 is one type of inhibitory Smad protein that negatively regulates activation and phosphorylation of Smad2/3. Overexpression of Smad7 could block activation of TGF‐β/Smad signalling while reduction of renal Smad7 could result in over‐activation of TGF‐β/Smad signalling and progression of renal fibrosis.^37^ In this study, we found AKBA increased the expression of Smad7 in vivo and in vitro experiments. These results indicated that AKBA has the effect of anti‐fibrosis by inhibiting the activation of Smad pathway.

In conclusion, our results revealed for the first time that AKBA could dose‐dependently improve renal interstitial fibrosis in vivo and in vitro, and the underlying mechanism may be involved with the inhibition of Klotho/TGF‐β/Smad signalling pathway. Our findings indicate that AKBA could be used as a novel candidate drug for renal interstitial fibrosis.

## CONFLICT OF INTERESTS

The authors declare no conflict of interest.

## AUTHOR CONTRIBUTIONS

S.S. involved in the conception and design of the experiments, M.L. and T.L. performed the animal research, P.S. and Y.Z. performed the in vitro research, L.L. and T.L. analysed the data, M.L. wrote the paper.
