# Supplementary material for: Acetyl‐11‐keto‐β‐boswellic acid ameliorates renal interstitial fibrosis via Klotho/TGF‐β/Smad signalling pathway
Source: J Cell Mol Med. 2018 Jul 28;22(10):4997–5007. doi: 10.1111/jcmm.13766 (PMC6156234; doi:10.1111/jcmm.13766)
Supplement: Supplementary file 1 [file JCMM-22-4997-s001.docx]

**Supporting information**

Table S1: Primers used in real-time RT-PCR.

Table S1. Primers used in real-time RT-PCR.

| **Gene** | **Forward primer (5'-3')** | **Reverse primer (5'-3')** |
| --- | --- | --- |
| TGF-β1 | TGGCCAGATCCTGTCCAAAC | GCGGGTGACCTCTTTAGCAT |
| Collagen I | TGTGCCACTCTGACTGGAAG | CGCCATACTCGAACTGGAATC |
| Collagen IV | ATGCACTCCAAGACTGCTCC | TTTGCCTTTGCAGACACAGC |
| α-SMA | GATGGTGGGAATGGGACAAA | GCCATGTTCTATCGGGTACTTC |
| GAPDH | AGGAGAGTGTTTCCTCGTCC | ATGGGCTTCCCGTTGATGAC |
